# Receiver-Initiated Handshaking MAC Based on Traffic Estimation for Underwater Sensor Networks ‡

**DOI:** 10.3390/s18113895

**Published:** 2018-11-12

**Authors:** Yuan Dong, Lina Pu, Yu Luo, Zheng Peng, Haining Mo, Yun Meng, Yi Zhao, Yuzhi Zhang

**Affiliations:** 1 School of Electronic Control, Chang’an Uiversity, Xi’an 710064, China; mengyun@chd.edu.cn (Y.M.); z1@chd.edu.cn (Y.Z.); 2 School of Computer Science and Computer Engineering, University of Southern Mississippi, Hattiesburg, MS 39406, USA; 3 Department of Electrical and Computer Engineering, Mississippi State University, Mississippi State, MS 39762, USA; yu.luo@ece.msstate.edu; 4 Computer Science Department, The City College of New York, New York, NY 10031, USA; zpeng@ccny.cuny.edu; 5 Oracle America Inc., Seattle, WA 98101, USA; mohaining0430@gmail.com; 6 School of Communication and Information Engineering, Xi’an University of Sience and Technology, Xi’an 700154, China; zhangyuzhinpu@hotmail.com

**Keywords:** underwater sensor networks, receiver-initiated MAC, adaptive data polling, traffic estimation

## Abstract

In underwater sensor networks (UWSNs), the unique characteristics of acoustic channels have posed great challenges for the design of medium access control (MAC) protocols. The long propagation delay problem has been widely explored in recent literature. However, the long preamble problem with acoustic modems revealed in real experiments brings new challenges to underwater MAC design. The overhead of control messages in handshaking-based protocols becomes significant due to the long preamble in underwater acoustic modems. To address this problem, we advocate the receiver-initiated handshaking method with parallel reservation to improve the handshaking efficiency. Despite some existing works along this direction, the data polling problem is still an open issue. Without knowing the status of senders, the receiver faces two challenges for efficient data polling: when to poll data from the sender and how much data to request. In this paper, we propose a traffic estimation-based receiver-initiated MAC (TERI-MAC) to solve this problem with an adaptive approach. Data polling in TERI-MAC depends on an online approximation of traffic distribution. It estimates the energy efficiency and network latency and starts the data request only when the preferred performance can be achieved. TERI-MAC can achieve a stable energy efficiency with arbitrary network traffic patterns. For traffic estimation, we employ a resampling technique to keep a small computation and memory overhead. The performance of TERI-MAC in terms of energy efficiency, channel utilization, and communication latency is verified in simulations. Our results show that, compared with existing receiver-initiated-based underwater MAC protocols, TERI-MAC can achieve higher energy efficiency at the price of a delay penalty. This confirms the strength of TERI-MAC for delay-tolerant applications.

## 1. Introduction

Underwater sensor networks (UWSNs) have demonstrated a wide range of important applications in oceanographic data collection, pollution detection, and marine surveillance [1,2]. Recently, UWSNs have spurred a great wave of efforts from the research community. However, the unique characteristics of UWSNs, such as their limited available bandwidth, long propagation delays, and high energy consumption, introduce great challenges to almost every layer of the network design [3,4]. In a network with shared media, medium access control (MAC) serves a fundamental role in network protocol stack coordinating multi-user communications. It is especially critical for acoustic networks with limited channel bandwidth, long propagation delays, and a constrained power supply.

To date, numerous MAC protocols, for example, [5,6,7], have been proposed and designed for UWSNs. Random access-based MAC protocols, such as those described in [8], are designed for simple implementation as well as robustness to adapt to dynamic networks. Nevertheless, their high collision probability limits their application in energy-constrained underwater networks. Alternatively, handshaking-based methods, e.g., [9,10], are a viable option for resolving collisions in the network system. The majority of existing handshaking MAC protocols for UWSNs have focused on how to handle the long propagation delays [11]. However, long preambles and the resulting high overhead on control packets have not been investigated thoroughly in the literature.

The handshaking MAC protocols can be generally classified into two categories. One is sender-initiated MAC, where the sender starts one round of handshakes by sending out a Request-to-Send (RTS) message to the intended receiver(s). Most of the existing handshake-based MAC protocols [9,10,11] are in this category. The other one is receiver-initiated MAC [12,13,14,15] where the receiver initiates the handshake process by sending a Request-to-Receive (RTR) message to poll data from intended senders. Despite improving the energy efficiency and enabling data aggregations, existing solutions to receiver-initiated-based handshake MAC protocols face great challenges in terms of efficient data polling. On the one hand, without knowing the status of neighboring senders, it is difficult for the receiver to decide when to poll data and how much data to request. On the other hand, the frequent information exchange between the sender and receiver leads to low efficiency in data communication. Following this concern, we propose a receiver-initiated handshaking MAC with adaptive data polling.

The contribution of our work is three-fold. First, we propose a traffic estimation-based receiver-initiated MAC (TERI-MAC). The receiver collecting data from neighboring nodes enables the data aggregation at the link layer, which benefits the data fusion and sensing process in underwater sensor networks. Second, we propose an adaptive data polling mechanism to solve the *when-to-poll* and *how-much-to-poll* problems in the receiver-initiated MAC protocols. With the assistance of online traffic estimation, TERI-MAC can be applied to networks with arbitrary traffic patterns while maintaining high energy efficiency and channel utilization for each user request. Third, extensive simulations have been conducted to evaluate the performance of TERI-MAC. It has been verified that TERI-MAC can achieve good energy efficiency and channel utilization by delaying the data requests at the receivers and adjusting the channel resource assignment among different senders.

The rest of this paper is organized as follows. We first discuss the applications and motivations of receiver-initiated MACs and enumerate challenges in Section 2. In Section 3, we survey the related research on the receiver-initiated MAC protocols and network traffic predictions. The network architecture is presented in Section 4. The details of TERI-MAC are elaborated in Section 5, with the adaptive data polling presented in Section 6. The traffic estimation technique is discussed in Section 7. We evaluate the performance of TERI-MAC in Section 8 and conclude this paper in Section 9.

## 2. Applications, Motivations, and Challenges

Receiver-initiated-based MACs have several advantages over sender-initiated-based handshaking protocols in data gathering applications of UWSNs, as will be discussed in this section. We also describe the major challenges in designing an efficient receiver-initiated-based MAC protocol.

### 2.1. Applications of Receiver-Initiated MAC

In applications such as underwater environment monitoring, offshore structural health monitoring (SHM), and oceanography data collection, environmental or structural information is frequently collected by the sensors in water and then forwarded to the sink nodes deployed on the ocean surface. The aggregated data flow in these applications naturally forms a tree network, where the sink nodes are the roots and the sensors are the leaves. The sensing pattern can either be periodic or have a varying rate depending on the environment (e.g., temperature and salinity, etc.) or human activity (e.g., oil drilling) [16]. When an interesting event is detected, bursty traffic from a short period of intensive observations may also be triggered. In addition, the traffic on a node is a combination of self-generated sensing data and the forwarding packets, resulting in traffic dynamics in both temporal and spatial dimensions. Therefore, we consider the data gathering tree network as an application that is preferable, yet challenging, to the receiver-initiated MAC protocols.

### 2.2. Motivations for Receiver-Initiated MAC

**Enabling Data Aggregation at the Link Layer.** In receiver-initiated MAC protocols, the receiving node requests and gathers data packets from surrounding neighbors at each round of communication. This feature enables data aggregation at the link layer which can significantly reduce the traffic overhead with the link layer packet fusion [17]. As assistance to the end-to-end data fusion, local information gathering is essential to support a fast response to the dynamics of a network and meanwhile, to extend the network‘s lifetime. In addition, receiver-initiated MAC protocols are preferable to the applications with network coding [18]. In order to successfully decode one data packet, the receiver may need multiple packets from different senders, since the coded packets generally diverge to the surrounding neighbor users. The scheme of one receiver invites multiple packets from different nodes in receiver-initiated protocols to fit the data gathering request of network coding applications.

**Significantly Reducing the Overhead on Control Messages.** For the purpose of achieving reliable communication, an acknowledgment (ACK) is needed for the receiver to inform the senders of successful data delivery. Since only one receiver is involved in parallel reservation with the receiver-initiated handshake, one ACK packet is enough to notify all the senders of the successful data reception in one round of communication. In contrast, in the sender-initiated-based protocols, each receiver in the parallel reservation is required to respond with an ACK message due to the one-to-many scheme, which results in extra energy consumption. When the overhead of control packets is comparable to the data packets in UWSNs owing to the long preamble problem [19], reducing the number of ACK messages could remarkably improve the energy efficiency of acoustic communications.

Based on the length of control and data packets listed in Table 1 [20], we summarized the control message overhead of the traditional sender-initiated handshaking without parallel reservation and the receiver-initiated method with parallel reservation, using the AquaSeNT OFDM (orthogonal frequency division multiplexing) Modem as an example. We assumed that five reservations were made on average with each round of parallel reservations.

- single-sender to single-receiver
  (RTS+CTS+ACK)/Data=0.66×3/1.15≈172%
- single-sender to multiple-receiver
  (RTS+5CTS+5ACK)/5Data=0.66×11/1.15/5≈126%
- multiple-sender to single-receiver
  (RTR+5ATS+ACK)/5Data=0.66×7/1.15/5≈80%
  RTSRequest-to-Send messageCTSClear-to-Send messageACKAcknowledgment messageRTRRequest-to-Receive messageATSAvailable-to-Send message

Besides energy saving with parallel reservation, the receiver-initiated communication scheme further reduced the network overhead roughly from 126% to 80%, illustrating a promising application for the energy-constrained UWSNs.

**Figure 1:**
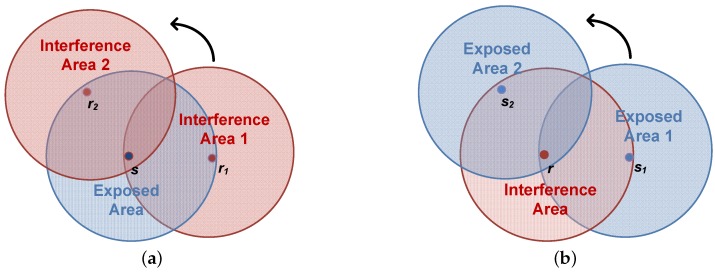
Interference and exposed areas in handshaking-based MAC protocols with parallel reservation. (**a**) Sender-initiated handshaking; (**b**) Receiver-initiated handshaking.

**Enhancing Collision Avoidance Capacity.** The interference area, marked as red in Figure 1, is the region within which the transmission of nodes will interfere with the reception process of the receiving node. In the communication with parallel reservation, either one sending node communicates with multiple receivers in sender-initiated-based methods, or one receiver requests data packets from multiple senders in receiver-initiated-based protocols. The interference area in Figure 1a changes while the sender communicates with different receivers in sender-initiated methods. In contrast, the receiver-initiated handshaking communication has a static interference area, as shown in Figure 1b, since only one receiver is involved. This static interference area enhances the collision avoidance for the time scheduling on the receiver side.

The exposed area where the exposed nodes lie is marked in light blue in Figure 1. In the traditional handshaking protocols, the exposed nodes that overhear the RTS message have to keep silent until hearing the corresponding Clear-to-Send (CTS) message. Even though the handshake mechanism helps to mitigate the exposed terminal problem, the nodes are still exposed for one propagation delay plus the transmission time of a control packet, neither of which is negligible in UWSNs. The exposed nodes in the receiver-initiated-based protocols, however, learn the fact that it is an exposed terminal when receiving an Available-to-Send (ATS) message immediately, since the RTR message has gone earlier. Therefore, the exposed terminal problem is mitigated in the receiver-initiated-based MAC protocols.

To summarize, the enabled data aggregation at the link layer, the significantly reduced overhead in the control message, and the enhanced collision avoidance capacity of receiver-initiated MAC protocols make them promising solutions for data gathering UWSNs.

### 2.3. Challenges in Receiver-Initiated MAC

Even though the receiver-initiated MAC design has its advantages over the traditional handshaking methods in many ways, it still faces great challenges in the implementation of data polling. Data polling is defined as the process whereby the receiver retrieves data from the neighboring senders. Since the receiver does not know the status of the neighboring senders, how to design an efficient data polling scheme becomes an open problem. The retrieval of data frequently results in energy waste when transmitting control messages, whereas slowing down the polling rate causes a long queuing delay because the data cannot be delivered timely. An efficient polling scheme needs to decide (1) when packets are available for the receiver to poll, and (2) how much data to request. For most of the receiver-initiated methods, *when* and *how much* are two fundamental challenges.

Furthermore, how to design a protocol that can adapt well to the dynamics and heterogeneity of network traffic is still yet to be explored. On the one hand, the number of queued packets at the link layer is a random variable that may vary with time and among different senders due to the combined effects of all the upper layers, e.g., network layer, transport layer, and application layer. On the other hand, it is not practical in underwater networks to approximate the traffic distribution with a large number of data samples. This is because of the limited computation and memory capacity of underwater sensors and the time-varying feature of network traffic. In TERI-MAC, the cumulative density function (CDF) inversion sampling technique is employed to pull a number of samples from the large history dataset and utilize the most recently observed data to determine the trend of the network traffic. In this way, a lightweight traffic estimation can be achieved to support the adaptive data polling mechanism in TERI-MAC.

## 3. Related Work

In this section, we review the existing receiver-initiated handshaking MAC protocols and survey the related work on the network traffic prediction.

### 3.1. Related Work on Receiver-Initiated MAC

The idea of receiver-initiated handshaking MAC is not new. Aiming to reduce the overhead of the handshaking procedure, Talucci et al. proposed MACA by invitation (MACA-BI) [13], a simplified version of multiple access collision avoidance (MACA) protocol by removing the transmission of RTS in the sender. In this method, a traditional *three-way* handshaking process is reduced to a *two-way* handshake. MACA-BI saves one round RTS message transmission; however, it suffers the hidden terminal problem in multi-hop networks.

Garcia-Luna-Aceves et al. designed the receiver-initiated multiple access (RIMA) protocol by reversing the handshaking process in wireless networks [14]. A *three-way* receiver-initiated handshaking process is employed in RIMA. The authors also extended the protocol to a version with dual-use polling (RIMA-DP), which allows both the polling and polled nodes to send data in one round of collision avoidance. Since no parallel reservation or packet train is implemented in the RIMA protocol, the overhead of the handshaking process is significant when it is applied to the underwater networks.

More recently, the receiver-initiated packet train (RIPT) protocol [15] was proposed for UWSNs. It employs both parallel reservation and a packet train to mitigate the long propagation delay problem of underwater acoustic channels and to enhance the handshaking efficiency. The RIPT utilizes a *four-way* RTR/SIZE/ORDER/DATA handshaking process. The SIZE message is a response to the RTR, based on which the receiver allocates the channel recourse among multiple senders. The new ORDER control message is introduced to schedule the packet train transmission of all involved sending nodes. Similar to RIMA-DP, the RIPT protocol also allows the receiver to transmit data in one round of collision avoidance. Without the prediction of when data will be available in the senders, the receiver simply starts one round of handshaking when it has data to transmit. When the network traffic features high dynamics, RIPT will suffer from poor handshaking efficiency.

The design of efficient data polling is a common challenge to all receiver-initiated MAC protocols in the literature. Without knowing the number of packets that have cumulated on the surrounding nodes, it is difficult for the receiver to decide a good time to initiate the next receiving request, especially with the dynamic traffic pattern. Therefore, in this work, we propose TERI-MAC, a receiver-initiated MAC based on traffic estimation. An adaptive data polling will enable stable energy efficiency in the networks with arbitrary traffic patterns. With the assistance of traffic estimation, the receivers in TERI-MAC are able to arrange the transmission scheduling on the RTR message without waiting for the ATS response from senders. The ORDER message can thus be removed in TERI-MAC to further reduce the overhead of the MAC protocol.

### 3.2. Related Work on Traffic Prediction

Traffic prediction is one of the major issues in measurement-based network control. An accurate prediction of the traffic load in the next time interval helps to provide a high quality of service (QoS) for multimedia applications [21] to improve resource allocation for cognitive radio systems [22] and to assist in capacity planning for Wi-Fi networks [23]. The traffic-related time series prediction problem has been extensively explored in both Ethernet and WiFi networks with different applications [23,24].

Traffic prediction algorithms are highly dependent on the traffic patterns and the statistical characteristics of traffics. As it is affected by the traffic aggregation and sampling (or smoothing) [25], the network traffic has a mix of self-similarity, short range, and long range dependencies [22]. If the network traffic is stable [26,27], we can model it as an autoregressive moving average (ARMA) process. However, if the network traffic is dynamic or its statistical properties change over time, ARMA cannot describe those variations very well. In this case, using an adaptive filter or a neural network [24] to predict the network traffic is a wise choice since they can continuously track the network changes by adjusting the weight of the filter (or neural network) in accordance with the instant traffic load. The neural network approach which can capture the non-linear nature of network traffic, however, is complicated to implement. The over-fitting problem in neural network forecasting also limits its applications to online prediction scenarios.

Traffic load in an arbitrary node of an underwater network, however, is quite different from the large-scale traffic of the Internet, which exhibits aggregation, self-similarity, and long-range dependence features. The traffic at the link layer tends to be more random and dynamic under the effect of all protocols at the upper layers. The packet generation pattern at the application layer and the control flow and reliability control at the transport layer as well as the paths selected at the network layer all affect the traffic pattern at the link layer. For this reason, we estimate the link layer traffic using statistical methods in this paper.

## 4. Network Architecture

**Figure 2:**
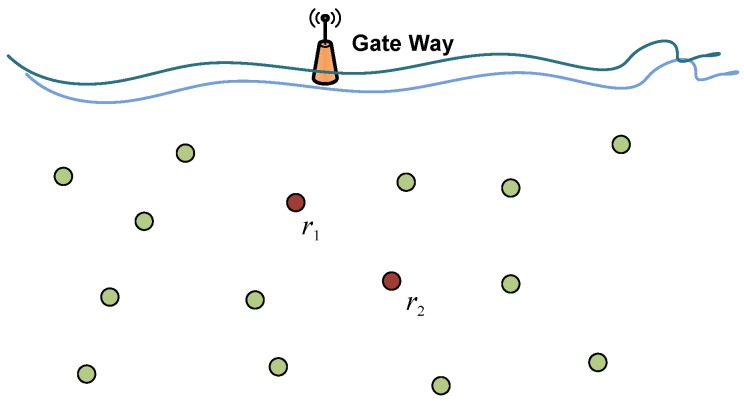
Data gathering network scenario.

Similar to the traditional sender-initiated handshaking MAC protocols, the receiver-initiated handshaking methods can be applied to a wide range of network applications. The parallel reservation in the receiver-initiated handshaking works particularly well in networks with specific data flow, e.g., data gathering networks. Figure 2 illustrates a data gathering network in which data from underwater sensors or vehicles is gathered to a surface buoy or a control center. This type of network has a wide range of applications, such as underwater environment monitoring as well as scientific exploration [28,29].

With parallel reservation, node $r_{2}$ in Figure 2 is able to collect information from its neighbor nodes. This data gathering scheme helps to reduce the channel competition and thus decreases the number of collisions among data packets. The receiver-initiated mechanism also enables packet merging at the link layer which further reduces the network traffic load. So, the receiver-initiated handshaking MAC protocols with parallel reservation are especially preferred in data gathering applications.

**Figure 3:**
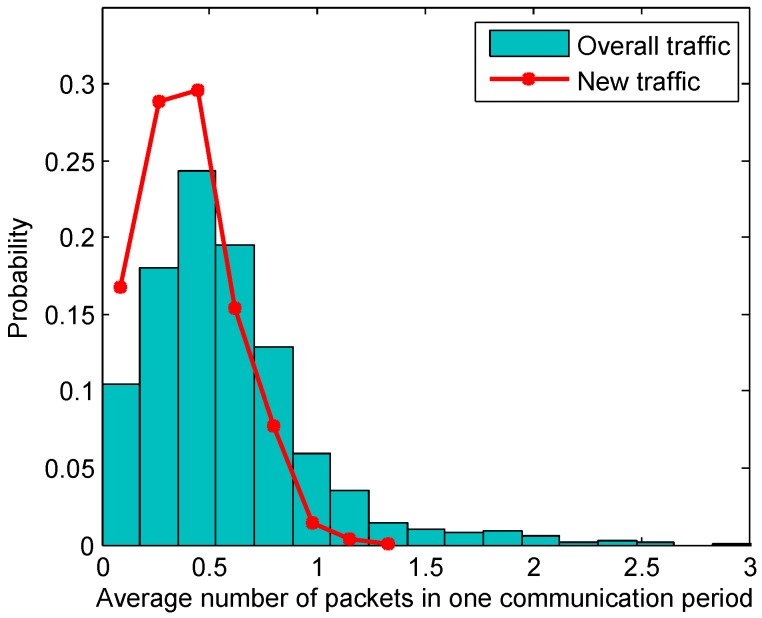
Distribution of the average number of packets on the link layer. The average data generation rate is 0.4.

In this paper, we assume the sensor nodes monitor the underwater environment (e.g., temperature, pressure, salinity) with periodical sensing. Because of the limited power supply and channel capacity, not every piece of data is reported, but only when some event is detected [30], i.e., temperature change, infusion target detection, and so on. This filtered information results in a random traffic generation rate in the underwater sensor network. We assume that the data packets on network nodes are generated independently with a given probability $p_{g}$ in each sensing period. For all nodes that act as relays, the total traffic, which includes its self-generated data and the relayed data, follows a Bernoulli Distribution. The traffic prediction problem is not trivial, since it is further affected by the behaviors of the transport and network layer protocols as well as the collisions and retransmissions on the link layer. Figure 3 shows that the resulting traffic has a much heavier tail than the traffic pattern on the application layer. For this reason, we study the link layer traffic from a probability point of view. The traffic estimation algorithm here updates the probability distribution of the number of packets, which supports the data polling for the receiver-initiated MAC protocols.

## 5. TERI-MAC Design

In this section, we first present the design of the TERI-MAC protocol in detail. The primary objective of TERI-MAC is to improve the network‘s energy efficiency and channel utilization in UWSNs. In TERI-MAC, the receiver needs to know the propagation delay to its neighbors, which could be measured through the classic *two-way* handshake approach that was tested in a sea experiment [31].

### 5.1. Description of TERI-MAC

**Figure 4:**
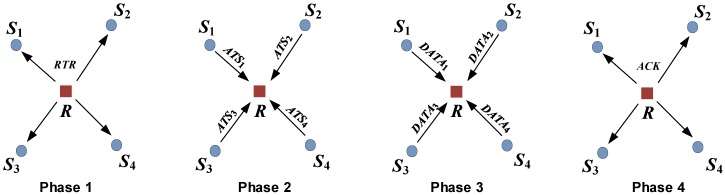
Four phases in traffic estimation-based receiver-initiated medium access control (TERI-MAC).

TERI-MAC employs a receiver-initiated *three-way* handshake. Specifically, it involves four phases, as shown in Figure 4.

**Figure 5:**
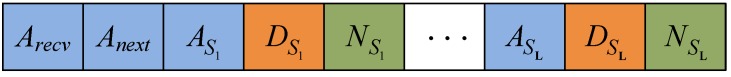
Format of the Request-to-Receive (RTR) message.

**Phase 1:** The receiver *R* starts a handshake process by sending out a Request-to-Receive (RTR) message when it intends to poll data from its neighbors. Here, the RTR message has two functions: to help the receiver request data from neighborhoods and to arrange the transmissions of Available-to-Send (ATS) packets from neighboring senders. It consists of the receiver’s ID, the next-hop receiver’s ID, the surrounding senders’ IDs, the transmission time of ATS in sequence, and the number of requested packets from each sender for the data transmission, as shown in Figure 5. By overhearing the RTR message, all the senders and potential interference nodes within the communication range are immediately notified of the upcoming data transmissions. No matter whether the neighboring node is scheduled to transmit the data or not, it will not interfere with the following data reception at the receiver. In this way, TERI-MAC is more effective at preventing the packet collisions caused by the hidden terminal phenomenon.

**Figure 6:**
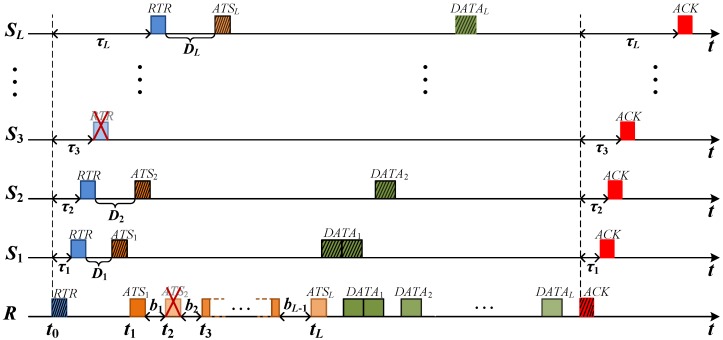
TERI-MAC transmission scheduling. Squares filled with and without oblique lines represent transmissions and receptions, respectively. The red cross indicates the occasional packet loss.

*RTR loss:* If the RTR message is not successfully decoded at the intended sender or potential interference node, we call it the RTR loss. Taking sender $S_{3}$ as an example, if the RTR is lost on $S_{3}$, the node will not be able to respond with ATS and thus, the handshake between *R* and $S_{3}$ will not be established. The time slots that have been assigned to sender $S_{3}$ will be wasted, degrading the channel‘s utilization. However, the handshake process and data transmissions continue on other senders that have successfully received the RTR message, as shown in Figure 6. Another negative effect of RTR loss is on the collision avoidance mechanism in TERI-MAC. Without the interpretation of the transmission scheduling carried on RTR, the node may not be aware of the following data receptions on the receiver *R*. Collisions are likely to happen if the node attempts to access a channel at the same time. In this case, overhearing the ATS message from other senders within the transmission range (i.e., $S_{1}$ and $S_{4}$ in Figure 4) can help mitigate the collisions caused by RTR loss.

**Figure 7:**
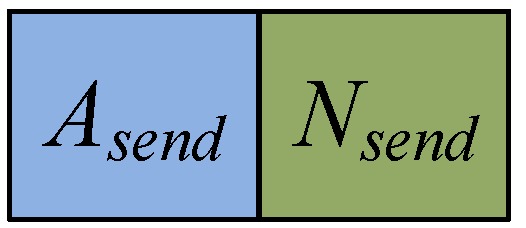
Format of the Available-to-Send (ATS) message.

**Phase 2:** The senders need to respond to the receiver with ATS messages to establish the data transmission sessions. In order to avoid collisions among ATS packets, the transmission time of the ATS packets should be staggered and follow the order scheduled in the RTR packet, which will be introduced in detail in Section 5.2. The ATS message includes the sender’s ID and the number of available packets during the time interval since the last round of data transmission, as shown in Figure 7. This information, as the input of the traffic estimation algorithm to be discussed in Section 7, helps the receiver to update the traffic distribution of each sender.

*ATS loss*: When the ATS message cannot be decoded, we call it ATS loss. Different from RTR loss, ATS loss does not affect the handshake process or the scheduled data transmissions, since the transmission is arranged in advance in the RTR packet. Figure 6 illustrates an example when the ATS from $S_{2}$ is lost on the receiver, *R*. The loss of the information about the number of available data packets on $S_{2}$ affects the traffic estimation to be performed on the receiver, *R*.

**Phase 3:** After establishing the handshakes between the receiver and the senders, the data transmission process begins. The senders send one or multiple data packets in a packet train according to the arrangement of the receiver. The time in the data transmission phase is divided into mini slots, which are assigned to the invited senders by the receiver in Phase 1, corresponding to $N_{S_{i}}$ in the RTR message. The length of time slot is equal to the transmission time of the DATA packets. Each sender sends out its DATA packets in the scheduled time if it is able to transmit. Since the slot allocation is based on the traffic estimation, which may not be accurate, the slots may be wasted if the invited sender has fewer packets to send or if some of the packets are not be transmitted in time if insufficient slots are assigned and are therefore queued at the sender. For this reason, TERI-MAC is more preferred in delay-tolerant applications. However, as long as we can get a good approximation of the traffic distribution of the senders, which is stationary, at least in the short term, TERI-MAC can achieve a desirable energy efficiency and network latency based on the adaptive data polling scheme which will be discussed in Section 6.

**Phase 4:** In the last phase of TERI-MAC, the receiver confirms the successful data reception by replying to an integrated acknowledgment (ACK) message to all senders, instead of individual ACKs from each receiver in the sender-initiated protocols. Considering the long preamble in acoustic modems, having fewer ACK packets will significantly reduce the power consumption and thus extend the network‘s lifetime. If the ACK message is lost on a sender, the node will not know whether the data reception is successful or not. The sender can either retransmit all involved data packets in the next round of data requests from the same receiver or dump those packets at the risk of potential data loss. In the simulation, we implemented the retransmission scheme to handle the occasional ACK loss to guarantee the delivery of data packets.

It is worth noting that, the guard time is generally required in most of the MAC protocols that utilize parallel transmissions to prevent unexpected collisions caused by an inaccurate distance measurement, a synchronization error or the drifting of acoustic nodes with an ocean current. In order to prevent collisions among ATS messages in Phase 2 and DATA packets in Phase 3, a guard time of 1 ms is added in the implementation of TERI-MAC.

Compared to the sender-initiated algorithms, one of the significant advantages of TERI-MAC is its energy efficiency. TERI-MAC conserves energy in three ways. Firstly, the receiver-initiated reservation is more effective at preventing data packet collisions since all the potentially interfering nodes of the receiver are no longer hidden and are informed at the very beginning of the handshaking process. Secondly, TERI-MAC employs parallel reservation and a packet train to reduce the overhead of handshaking control messages. Furthermore, the number of ACK packets is reduced dramatically compared to the sender-oriented protocols.

### 5.2. Arrangement of ATS Transmission

The ATS packets from different senders should arrive at the receiver in a staggered manner to prevent ATS collisions, as introduced in Phase 2 of Section 5.1. Based on the receiver-initiated protocols, each receiver in TERI-MAC needs to arrange the transmission of ATS packets for these invited senders in advance in the RTR packet. By optimally scheduling the ATS transmission, one can get a minimal ATS reception time while improving the utilization of the acoustic channel.

Now, assume that there are *L* senders, denoted by a set $S=[S_{1},S_{2},\ldots ,S_{L}]$. Each sender $S_{i}$ transmits an $ATS_{i}$ packet to the common receiver *R*, and the propagation delay between *R* and $S_{i}$ is denoted by $\tau_{i}$. At time $t_{0}$, the receiver *R* transmits an RTR packet and at time $t_{i}$, the receiver *R* receives the $ATS_{i}$ from the sender $S_{i}$ successively, where i∈{1,…,L}. The transmission times of RTR and ATS are denoted by $T_{RTR}$ and $T_{ATS}$, respectively, and the time difference between the RTR reception and the $ATS_{i}$ transmission on $S_{i}$ is labeled $D_{i}$. Let $b_{i}$ (i∈{1,…,L−1}) denote the reception interval among $ATS_{i}$ and $ATS_{i+1}$, as shown in Figure 6.

In order to avoid collisions among ATS messages, each sender $S_{i}$ should wait for an appropriate time difference $D_{i}$ to make the $b_{i}\geq 0$ (i.e., no ATS overlapping at the receiver). Based on the fact that the differences in propagation delays to different senders are marginal compared to the length of control packets considering the long preamble in acoustic modems, it is highly possible to ensure $b_{i}=0$ and to obtain a minimum total time for all ATS receptions. In this case, according to the propagation delay between the receiver and the sender $S_{i}$, we have

(1) $$D_{i}=t_{i}-\left(t_{0}+T_{RTR}\right)-2\tau_{i},\;i\in \left\{1,\ldots ,L\right\}.$$

If $ATS_{i}$ is the $l^{th}$ of *L*ATS packets arriving at the receiver, then Equation (1) can be written as

(2) $$D_{i}=T_{m}-\left(L-l\right)T_{ATS}-\left(t_{0}+T_{RTR}\right)-2\tau_{i},\;i\in \left\{1,\ldots ,L\right\}$$

where $T_{m}$ is the local time at the receiver receiving the last ATS packet, i.e., $T_{m}=\max\left\{t_{i}\right\},i\in \left\{1,\ldots ,L\right\}$. Then, the goal of minimizing the total time for all ATS packets receptions is equivalent to minimizing $T_{m}$.

Since $D_{i}\geq 0$, from Equation (2), we can get

(3) $$T_{m}\geq \left(L-l\right)T_{ATS}+\left(t_{0}+T_{RTR}\right)+2\tau_{i},\;i\in \left\{1,\ldots ,L\right\}.$$

By observing Equation (3), we find that $\left(t_{0}+T_{RTR}\right)$ is a constant. So, in order to minimize $T_{m}$, we have to add the smallest $\left(L-l\right)T_{ATS}$ to the largest $\tau_{i}$. More specifically, an ATS packet from a sender with the largest propagation delay should be the last to arrive at the receiver. Similar rules apply to other senders. Therefore, an ATS packet from a sender with a larger propagation delay (larger τ) should come after the one from a closer sender (smaller τ) and vice versa.

If we sort senders in the set S by the propagation delay τ in an ascending order, i.e., sender $S_{L}$ has the largest propagation delay $\tau_{L}$, and Equation (3) can be rewritten as

(4) $$T_{m}\geq \left(L-i\right)T_{ATS}+\left(t_{0}+T_{RTR}\right)+2\tau_{i},\;i\in \left\{1,\ldots ,L\right\}.$$

Therefore, the minimum $T_{m}$ is

(5) $$T_{m}=\max_{i}\left\{\left(L-i\right)T_{ATS}+\left(t_{0}+T_{RTR}\right)+2\tau_{i}\right\},\;i\in \left\{1,\ldots ,L\right\}.$$

Finally, the receiver calculates $t_{i}$ by $t_{i}=T_{m}-\left(L-i\right)T_{ATS}$ and the sender delay $D_{i}$ by $D_{i}=t_{i}-t_{0}-2\tau_{i}$ for each sender and then attaches the time difference $\left[D_{1},D_{2},\ldots ,D_{L}\right]$ and the MAC addresses of the corresponding senders to an RTR packet. After the RTR reception, sender $S_{i}$ is delayed for $D_{i}$ before replying to its ATS.

From the above descriptions, we know that the knowledge of the receiver’s local time is not necessary on the sender side, rather, the time difference $D_{i}$ is sufficient for the scheduling of ATS transmission, which allows it to operate in a non-synchronized UWSN.

## 6. Adaptive Data Polling for TERI-MAC

The data polling mechanism is one fundamental problem for the receiver-initiated MAC protocols since the receiver that starts the communication has little information on the transmission requests of senders. More specifically, the data polling problem includes two issues, one is when to poll data, the other is how much data to poll. In this section, with the assistance of link layer traffic estimation, we discuss and propose an adaptive data polling algorithm to solve these two issues. Based on the historical feedback of the packet number on the sender side, the receiver can estimate the traffic from different senders. Traffic estimation can help the receiver to poll data in time and to avoid over-requesting.

### 6.1. When to Poll Data

In most of the receiver-initiated methods, due to the lack of knowledge of the current status of the intended senders, e.g., having packets to send or not, on the receiver side, receivers usually blindly decide when to poll data from the surrounding senders. Frequent data polling results in high energy consumption if the sender has few packets to transmit. The parallel reservation and packet train lose effectiveness when the data is polled too frequently. Conversely, if receivers do not request data in time, long delays will be introduced into the data transmission, which is also not acceptable in most applications. Therefore, a receiver needs to adjust the frequency of data polling in order to get an appropriate tradeoff between energy efficiency and communication latency.

In UWSNs with a constrained power supply, high energy efficiency is preferred to extend the network’s lifetime. In order to achieve a controllable performance, we set up a threshold of the control packet overhead $E_{th}$, which is defined as the total power consumption of the control packets during the data transmission during one round handshake, and the average hop-by-hop communication delay $D_{th}$, which is defined as the average delay for data packets waiting for transmission. For each node $Q_{i},i\in \left\{1,\ldots ,N\right\}$, where *N* is the total number of nodes in the network, $Q_{i}$ will start the data request in three cases, as follows.

- **Case 1:** If $Q_{i}$ is the next-hop destination of the current active receiver, $Q_{i}$ will start the data polling as soon as it can so that the packets can be forwarded to the final destination smoothly in the multi-hop communications. As shown in Figure 5, the RTR message including the $A_{next}$ information notifies the successive node of the coming data reception. It is worth noting that the $A_{next}$ does not indicate the dependency of TERI-MAC on specific routing protocols. When a new packet is generated or a forwarding packet is received, it is temporally stored in a queue before being sent out. The node also records the generation or reception time and the next-hop receiver’s ID, $A_{next}$ for each packet. The $A_{next}$ information can be obtained from the routing header. When a node receives an RTR request from $A_{next}$, all the packets with a matched next-hop ID have the chance to be transmitted in a group. Although TERI-MAC can work with most of the existing routing protocols, clustering-based routing enables better data aggregation, allowing TERI-MAC to achieve higher throughputs and better energy efficiency.
- **Case 2:** $Q_{i}$ starts the handshake if the estimated energy efficiency $E_{est(i)}$, which is the power consumption of control packets over the estimation of data transmission, is below the defined threshold of performance $E_{th}$. Here, $E_{est(i)}$ is estimated by the receiver based on the traffic estimation which is introduced in the next section. In this way, a baseline energy efficiency $E_{th}$ can be achieved in TERI-MAC.
- **Case 3:** $Q_{i}$ requests data from its neighbors when the time interval $D_{i}$ since the last communication exceeds the delay threshold $D_{th}$. In networks with a low traffic load, the waiting time to cumulate packets and achieve the desired energy efficiency becomes too long to be acceptable, since the out-of-date sensing information may become useless even if the destination is received successfully. The hop-by-hop communication delay threshold $D_{th}$ can guarantee the maximum delay performance. In most cases, we can achieve preferable energy efficiency with a tolerable amount of delay.

### 6.2. How Much Data to Poll

The second challenge of the data polling problem is to decide how much data to request from each sender. Since the receiver has no prior knowledge regarding the number of data packets cumulated on the intended senders, the receiver has trouble determining how many packets to request from senders without any extra communication. By assigning more channel resources to the sender, a higher packet delivery coverage and thus, a shorter communication delay can be achieved at the cost of lower efficiencies for both energy and channel utilization. The receiver can improve the energy and channel efficiency by assigning few transmission slots to senders, such that the assigned slots have less chance of being wasted, which, however, may result in an extra queuing delay when the cumulated packets cannot be sent out in a timely manner. Therefore, a good estimation of the probability distribution of the senders’ traffic is especially important. With the assistance of a good traffic predictor, the receiver is able to guarantee an overall packet delivery ratio. More information about the traffic estimation is introduced in Section 7.

In an underwater network, deployed as Figure 2 for example, the receiver node initiates the handshaking by requesting data from its neighbors. The traffic from different senders may vary since it includes both the self-generated data and the packets forwarded for other nodes, according to the network topology and the routing protocol. The traffic in node $Q_{i},i\in \left\{1,\ldots ,N\right\}$ would follow the binominal distribution, and we have

(6) $$TRA_{Q_{i}}∼\sum_{j=1}^{N_{Q_{i}}}B\left(T_{j},p_{gj}\right)$$

where $N_{Q_{i}}$ represents the number of nodes that have packets to travel through node $Q_{i}$ which may vary with time and depends on the network topology and routing algorithm. $T_{j}$ is the time interval between two successive rounds of data requests, and $p_{gj}$ is the self-generated average data rate.

- **The delivery percentage $P_{\mathrm{del}}$:** When the number of packets in the polled node follows a given distribution F(x), there is a certain probability $P_{del}$ that the assigned slots $T_{S}$ will be enough to transmit all of the queued packets:
  (7) $$P_{del}=\int_{0}^{T_{S}}F\left(x\right)dx.$$

Since the number of packets is a random process, there is no way for the receiver to predict an exact number without error. However, based on traffic distribution knowledge, the receiver can assign time slots for each sender in order to guarantee a predetermined delivery percentage $P_{del}$.

By adjusting percentage $P_{del}$, we can achieve a tradeoff between channel resource utilization and communication delay. A high $P_{del}$ increases the chance that a packet will delivered in the current round of communication; however, it also increases the probability that the channel resource be wasted because of the overassigned time slots. A low $P_{del}$, on the contrary, leads to a high average delay, which is not profitable for the network communications. Choosing an appropriate $P_{del}$ is important to TERI-MAC, as will be discussed later in Section 8.1.

## 7. Traffic Estimation for TERI-MAC

During the handshaking process, the receiver is informed of the average packet number within the time interval between two successive data requests, serving as input information for the traffic estimation. Now, let $x_{i}$ denote the number of packets and $X_{M}=\left[x_{1},x_{2},\ldots ,x_{M}\right]$ be the set of the samples, where *M* is the size of $X_{M}$. A standard method for an approximating distribution is the empirical distribution of the observed data from all of the historical records, e.g., the sample set $X_{M}$. We can get a better estimation as the sample size *M* increases. However, because of the traffic dynamics in the time domain and the constrained computation and memory capacity in the underwater nodes, we can only expect a sparse dataset for a rough traffic model approximation.

On the one hand, a larger sample size leads to a more accurate traffic estimation and a more efficient channel allocation for the TERI-MAC. On the other hand, large datasets slow down the adaptation of the traffic model to the temporal variation of the link layer traffics and aggravate the computation overhead of underwater nodes. Instead of keeping all the history samples, $\{x_{i}\},i=\;1,\ldots ,M$, we utilize the CDF Inverse Sampling technique to generate a set of samples $\left\{{\hat{x}}_{i}\right\},i=1,\ldots ,M^{\prime }$ of a constant size $M^{\prime }$ where $M^{\prime }<M$ from the traffic distribution F(x) is a representation of the large dataset.

- **CDF Inverse Sampling** [32]: Let G(x) be the cumulative distribution function (CDF) of a random variable *x* which has the probability density function (PDF) F(x). If random variable *y* comes from a uniform distribution U(0,1), then the random variable $\hat{x}=G^{-1}\left(y\right)$ follows the same distribution as *x*. In other words, a set of samples $\left\{\hat{x_{i}}\right\}$ becomes a good representation of random variable *x* as they have the same distribution (F(x)).

CDF Inverse Sampling is a simple but effective method when the distribution is univariate and the inverse CDF is easy to obtain. In many cases, when inverse CDF cannot be solved analytically, we can approximate the true CDF with a piecewise linear function based on the sample set from the distribution [33].

In order to determine the trend of traffic when it varies, the most recent *K* records $\left\{x_{M-K+1},\ldots ,x_{M}\right\}$ are used to approximate the traffic distribution together with samples $\left\{{\hat{x}}_{1},\ldots ,{\hat{x}}_{M^{\prime }}\right\}$. The sampling window of size *K* represents the most recent trend of the network’s traffic, while the resampled data $\left\{{\hat{x}}_{1},\ldots ,{\hat{x}}_{M^{\prime }}\right\}$ from the preceding records presents the historical information of the traffic distribution. With the small dataset $\left\{{\hat{x}}_{1},\ldots ,{\hat{x}}_{M^{\prime }},x_{M-K+1},\ldots ,x_{M}\right\}$, we can then estimate the traffic distribution by leveraging an affordable computation, e.g., the kernel density estimation method.

When the new record $x_{M+1}$ arrives, the sampling window moves forward and leaves the sample $x_{M-K+1}$ out of the window. We also need to resample the historical records $\left\{x_{M-K+1},{\hat{x}}_{1},\ldots ,{\hat{x}}_{M^{\prime }}\right\}$ to keep a non-increasing dataset. To reduce the frequent resampling process in the traffic estimation, a simplified procedure is illustrated in Algorithm 1. A sampling window with a changing size is employed. The size of the sampling window $S_{WIN}$ increases with the new arrival records. When $S_{WIN}\geq K$, all the records in the sampling window are moved out and merged to the data set $\left\{{\hat{x}}_{1},\ldots ,{\hat{x}}_{M^{\prime }}\right\}$, and the set is resampled to get a new representation for the historical distribution.

**Algorithm 1** Traffic estimation in TERI-MAC.**while** new record $x_{M+1}$ comes **do**    **if** $S_{WIN}\geq K$ **then**        Move out $x_{M-K+1},\ldots ,x_{M}$ in the sampling window;        Resample from $\left\{{\hat{x}}_{1},\ldots ,{\hat{x}}_{M^{\prime }},x_{M-K+1},\ldots ,x_{M}\right\}$    **end if**    Add $x_{M+1}$ in the sampling window;    Build PDF with $\left\{{\hat{x}}_{1},\ldots ,{\hat{x}}_{M^{\prime }}\right\}$ and records in sampling window;**end while**


**Figure 8:**
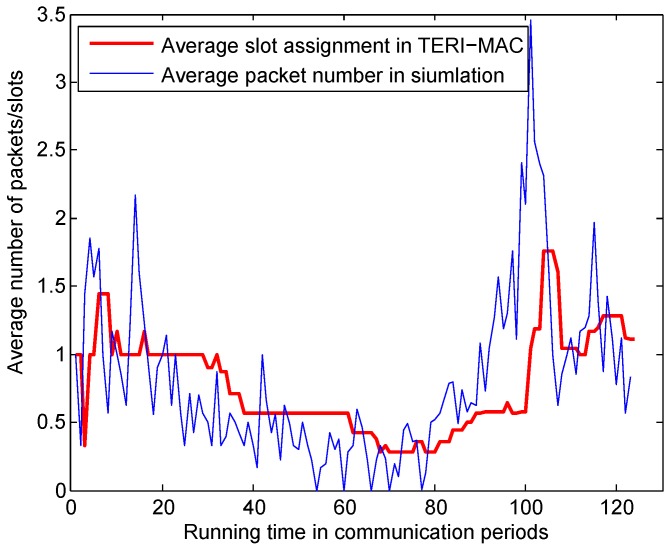
Adaptive slot assignment in TERI-MAC with a varying traffic load.

Depending on the traffic estimation designed in Algorithm 1, the receiver estimates how much data to request from each sender and assigns time slots for their data transmissions. We simulated an experiment to show the average traffic of one sender and the channel resource assignment with traffic estimation at the receiver side. In this simulation, we tested the ability of the traffic estimation mechanism used in our work to capture the dynamic traffic and perform accurate predictions. A sequence of random traffic was generated following a Bernoulli distribution and the average traffic load varied from 0 to 2 to manipulate the traffic load variation over time in real applications. The traffic load was the average number of data packets generated per time slot. In addition, we set a sampling window size of K=10 and resampled $M^{\prime }=5$ from the historical records each time. Figure 8 illustrates that even though a lag existed from the moment of the traffic change and the slot assignment, the traffic estimation followed the trend very well. Furthermore, we computed the channel utilization by calculating the ratio of the time slots utilized for data transmission over the total amount assigned by the receiver. Based on the premise of $P_{del}=50\%$, the simulation results verified that an overall 88% channel utilization was achieved when trying to assign the slots to guarantee a 50% probability of packet coverage in the transmission. This simulation verifies the efficiency of the traffic estimation mechanism.

## 8. Simulation and Analysis

This section describes the evaluation of the performance of TERI-MAC via simulations. The simulation platform was Aqua-Sim [34], an NS-2 based underwater network simulator. Three performance metrics were used as follows:

- **Energy Efficiency:** The energy efficiency was evaluated in terms of the relative control message overhead, defined as the overhead of control messages divided by that of data packets in one round of data communication.
- **Channel Utilization:** The ratio of the time slots utilized for data transmission in the data communication phase (Phase 3 in Figure 4) over the total amount assigned by the receiver.
- **Hop-by-Hop Communication Delay:** The average queuing delay for the data transmission, i.e., the time period between when the original data packet is generated or the relay data is received and when the packet is transmitted to the next-hop receiver.

The simulation results verified the enhanced energy efficiency, delivery delay, and channel utilization of TERI-MAC with the adaptive data polling mechanism. We also compared TERI-MAC with RIPT [15] which is briefly introduced in Section 3.1. All results presented in this section are the average outcomes of multiple tests.

**Figure 9:**
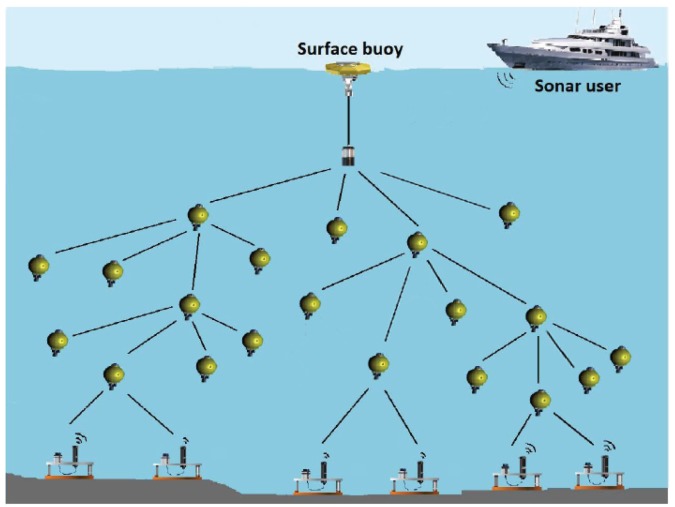
Tree topology in the network simulation.

We evaluated the performance of TERI-MAC in a tree network topology, a typical topology for underwater sensing data collection. In this topology, 20 sub-sea nodes collect and forward data to the sink node on the surface of the ocean, as illustrated in Figure 9. For each branch, we let four intended senders transmit to a common receiver. In addition, the average distance between neighboring nodes was 1 km (uniformly distributed between 800 m and 1200 m). The maximum transmission range and the maximum transmission power of each node were set as 1.5 km and 20 watts, respectively. The size of data packets was 250 bytes.

The overall available channel bandwidth in the simulation was 5 kHz (from 20 kHz to 25 kHz). We implemented the classic frequency-dependent absorption model and the shallow water noise model presented in [35]. Aqua-Sim [34] includes a default bit error model where each bit in a receiving packet has an independent chance of having an error. Whether a packet can be successfully decoded depends not only on the bit error rate (BER) but also on the error correction coding, which is not implemented in the current version of Aqua-Sim. Therefore, the BER in the simulation was set to zero in the evaluation of both TERI-MAC and RIPT MAC. We expected a performance degradation for both protocols if the BER was remarkable. Although the BER was not considered, the channel in our simulation was not error-free. Strong attenuation strong or high noise in the environment would cause the signal-to-noise ratio (SNR) of the receiving packet to be weak, leading to a packet error. This, to some extent, allowed us to simulate the effect of physical error (even if not colliding) on the performance of the MAC protocols.

### 8.1. Performance Evaluation

We first evaluated the energy efficiency performance of the TERI-MAC system, which is controlled by the parameter, $E_{th}$, which represents the target energy efficiency in the Algorithm 2. Be aware of the traffic models of all neighbor senders, the receiver that initiated the handshaking communication was able to adjust the frequency of data polling to reach the required energy efficiency. The traffic estimation enabled TERI-MAC to adapt to any traffic pattern. If the traffic load was relatively low, the waiting time increased to allow more packets to be accumulated at the senders. For high traffic loads, the receiver would request the data more frequently after achieving the desired energy efficiency.

**Figure 10:**
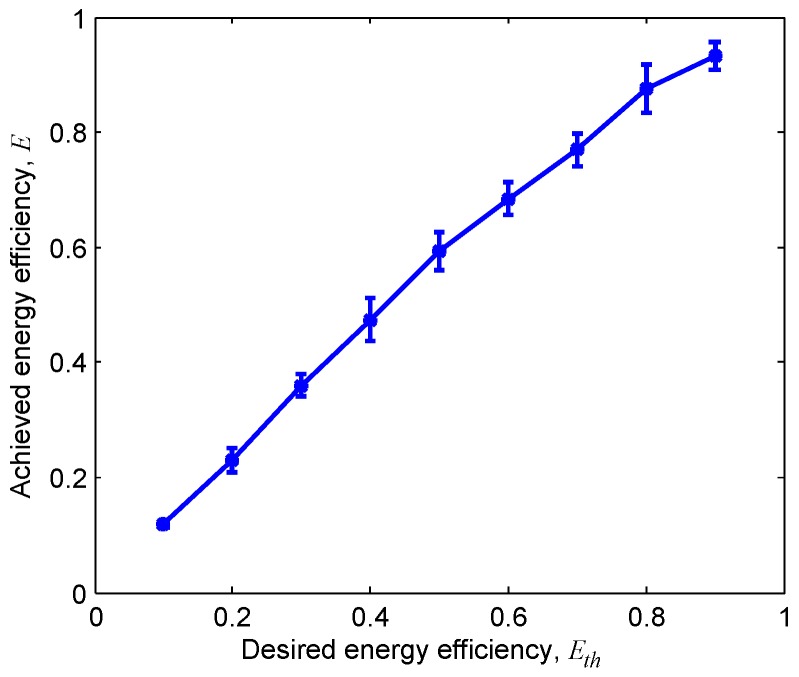
The achieved energy efficiency with respect to different parameter settings ($E_{th}$) in TERI-MAC.

The energy consumption of the control and data packets in our simulations was calculated based on the AquaSeNT OFDM Modems presented in Table 1. The average traffic generation rates of four senders involved in the simulation were set as λ=0.02,0.03,0.05,0.1, respectively. Figure 10 illustrates that TERI-MAC was able to achieve the desired energy efficiency by adjusting parameter $E_{th}$ from 0.1 to 0.9. In other words, the good consistency between the desired $E_{th}$ and the achieved *E* verified the effectiveness of both the energy efficiency control and the traffic estimation procedure in TERI-MAC.

**Figure 11:**
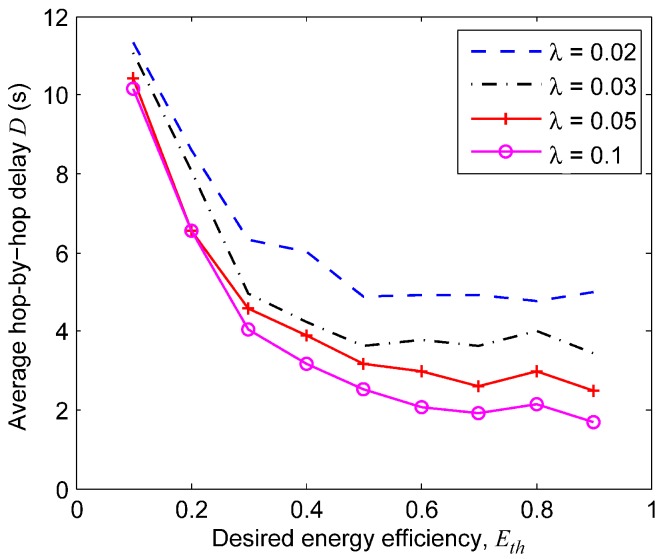
Tradeoff between energy efficiency and the hop-by-hop delay performance.

As introduced in the problem of when to poll data in Section 6.1, the frequency of data polling is a key factor that affects energy efficiency and delays performance, and thus, Algorithm 2 is was proposed to decide when to poll data based on the threshold of the control packet overhead $E_{th}$. Figure 11 shows the tradeoff between the energy efficiency and the average hop-by-hop communication delays of the four senders. The simulation results revealed two facts as follows: (a) The delays significantly increased when the target control packet overhead $E_{th}$ decreased from 0.3 to 0.1 for all senders. In other words, a relatively low $E_{th}$ resulted in a longer hop-by-hop delay. (b) For senders with different traffic loads λ, a larger λ resulted in a longer average hop-by-hop delay. We found that the protocol worked with high efficiency and acceptable delay when $E_{th}=0.2$. For delay-sensitive applications, a control packet overhead as low as 0.3 can be achieved in TERI-MAC.

**Algorithm 2** When to poll data.**if** $A_{Q_{i}}==A_{next}$ **then**      % $A_{Q_{i}}$ denotes the address of the node $Q_{i}$    Poll data when current handshaking ends;**else**    **while** $E_{est(i)}\geq E_{th}\;\&\;max\left(D_{j}\right)\leq D_{th}$ **do**   % where *j* is the index of intended senders around $Q_{i}$        Waiting time ++;    **end while**    Poll data if channel idle;**end if**


**Figure 12:**
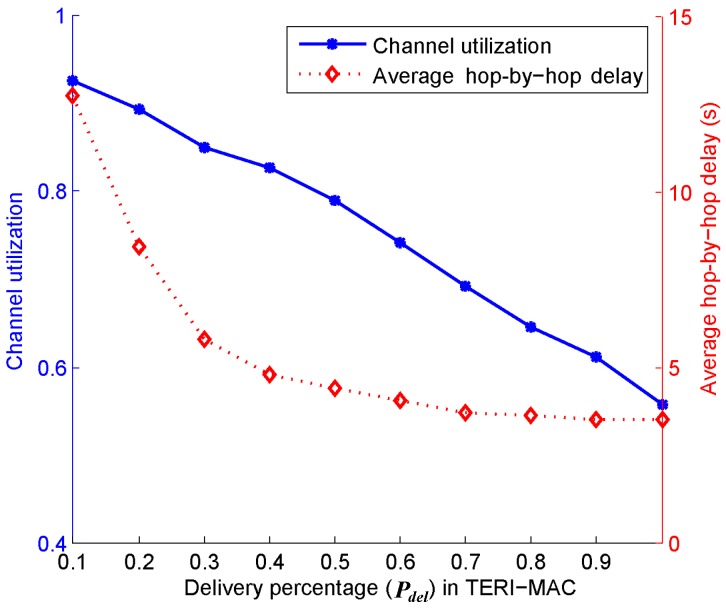
Tradeoff between channel utilization and the hop-by-hop communication delay performance.

Next, we conducted simulations to verify the efficiency of Algorithm 1, which solves the how much data to poll problem. As introduced in Section 6.2, the transmission slot allocation among senders based on the traffic estimation acts as a tradeoff between channel utilization and delay performance by adjusting the delivery percentage $P_{del}$ from 0.1 to 1. Figure 12 demonstrates that the channel utilization linearly decreases with the increase of $P_{del}$, presenting a reduced delay in an inverse proportional way. The simulation results revealed that a $P_{del}$ within [0.40.5] can achieve an acceptable channel utilization and delay.

### 8.2. Performance Comparison

To verify the advantages of the proposed system, we compared TERI-MAC with RIPT. A brief introduction on RIPT was presented in Section 3.1. Furthermore, we also ran RIPT with the traffic predictor to validate the effectiveness of the adaptive data polling mechanism based on the traffic estimation.

**Figure 13:**
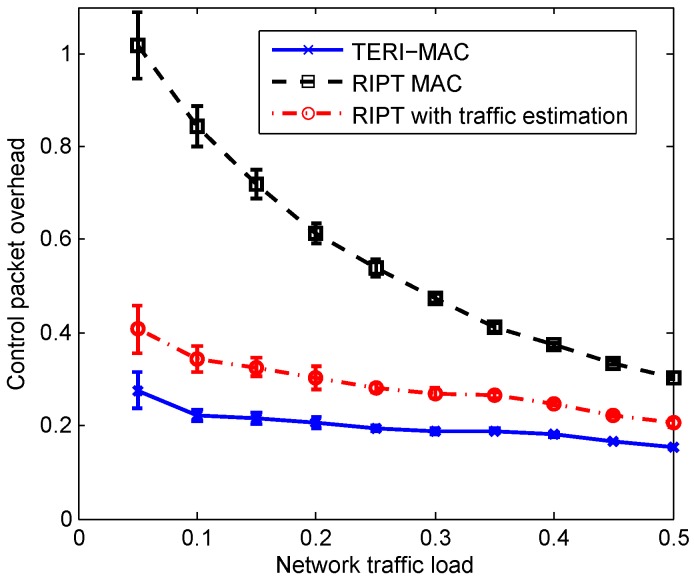
The overhead of the control packet with respect to the network traffic load.

**Figure 14:**
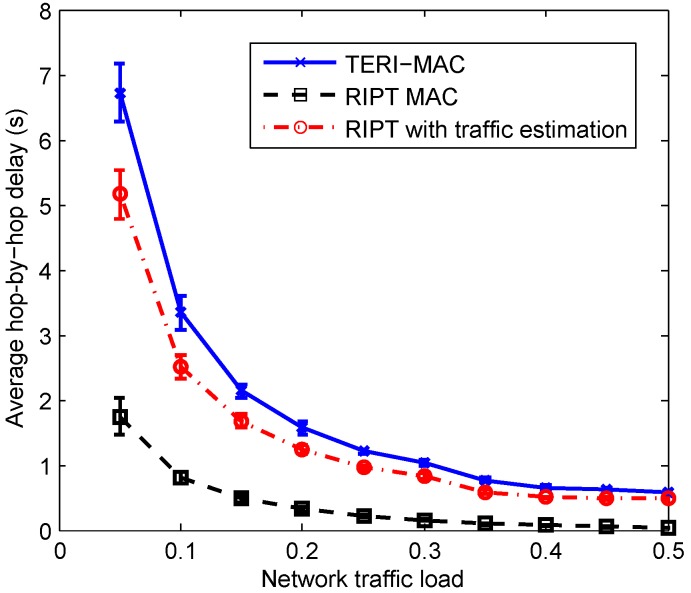
The hop-by-hop communication delay with respect to the network traffic load.

Figure 13 and Figure 14 demonstrate the performance of three methods (i.e., TERI-MAC, RIPT MAC and RIPT with traffic estimation) in terms of the energy efficiency and hop-by-hop communication delay. As revealed in Figure 13, TERI-MAC achieved the best energy efficiency as revealed in Figure 13, at the cost of a relatively long hop-by-hop communication delay, as shown in Figure 14. In the TERI-MAC simulation with $E_{th}=0.2$, a roughly stable control packet overhead was achieved with a wide range of network traffic loads, which was much lower than the RIPT MAC, especially at low traffic loads. However, a longer hop-by-hop communication delay was introduced in the TERI-MAC, due to the less frequent data polling when the network had a light traffic load. In addition, we observed the heavy reliance of the performance of RIPT on the network traffic load. When the traffic load was low, there were only few data packets available in the senders for each handshaking communication. This overfrequent data polling in RIPT MAC resulted in a relatively high control packet overhead and thus a poor energy efficiency. The efficiency performance improved at a high traffic load but was still worse than that of TERI-MAC.

We also implemented the proposed traffic estimation for RIPT MAC in order to verify the efficiency of the traffic estimation scheme. The modified RIPT achieved a significant improvement in energy efficiency, as shown in Figure 13. With an estimation of the number of packets at each sender, the polling receiver was able to detect the low traffic load of the network and tended to slow down the handshaking process to accumulate packets. Even though a longer delay resulted from the adaptive data polling scheme, the substantial energy efficiency improvement is more promising for its use in power constraint UWSNs. The efficiency enhancement to RIPT reveals the effectiveness of our traffic estimation-assisted adaptive data polling scheme for receiver-initiated MAC protocols.

## 9. Conclusions

In this paper, we designed a new receiver-initiated system based on traffic estimation, TERI-MAC to achieve efficient communications for UWSNs. In TERI-MAC, receivers replace the role of senders in conventional protocols to initiate the handshaking process. The parallel reservation, shared control packets, and enabled link layer data aggregation in TERI-MAC significantly improve the energy efficiency of UWSNs compared to the long preamble in acoustic modems.

The unique challenges faced in the receiver-initiated MAC approaches, that is, to decide when to poll and how much data to poll, were solved by adopting traffic estimation in our paper. By employing the adaptive data polling mechanism, the receiver in TERI-MAC can initiate handshakes timely to reduce the packet queuing delay while constraining the energy consumption on transmitting control packets. The simulation results verified that our traffic estimation adapts well to dynamic traffic loads in the network, and the adaptive data polling mechanism in TERI-MAC results in stable energy efficiency with varying traffic patterns.

In the meantime, we carried out extensive simulations and evaluated the energy efficiency, the channel utilization, and the hop-by-hop delay performance of TERI-MAC. The results demonstrate that by adjusting the frequency of data polling, the desired energy efficiency can be achieved. A trade-off between channel utilization and delay performance is also expected in TERI-MAC. Moreover, the comparative experiments proved that the performance of TERI-MAC with traffic estimation outperforms RIPT, a representative receiver-initiated-based underwater MAC protocol. Therefore, we believe that TERI-MAC is a promising MAC solution for delay-tolerant underwater applications.

## Figures and Tables

**Table 1:** Overhead of control message (6 bytes) and data packet (250 bytes) with different acoustic modems.

Modem Type	Data Rate	Preamble (s)	Control Packet Length (s)	Data Packet Length (s)
Benthos ATM-88X Modem	800 bps (Standard)	≈1.5	≈1.56	≈4.06
	2.4 Kbps (Highest)		≈1.52	≈2.35
AquaSeNT OFDM Modem	3.045 Kbps	0.49	0.66	1.15
WHOI Micro Modem	80 bps (Standard)	0.87	1.47	25.87
	300–5000 bps (PSK mode)		0.88–1.03	1.27–7.54

## Abbreviations

$A_{recv}$	Address of the active receiver that sends the RTR
$A_{next}$	Address of the next-hop receiver of $A_{recv}$
$A_{S_{i}}$	Address of the sender *i* that is invited by the receiver, where i∈{1,…,L}
$D_{S_{i}}$	Time schedule of ATS transmission for sender *i*
$N_{S_{i}}$	Number of DATA packets assigned to sender *i* for the following data transmission
$A_{send}$	Address of the sender that responds to the ATS
$N_{send}$	Average number of DATA packets since the last transmission
